# State-Level Variation in Medicaid Managed Care Enrollment and Specialty Care for Publicly Insured Children

**DOI:** 10.1001/jamanetworkopen.2023.36415

**Published:** 2023-10-05

**Authors:** Ju-Chen Hu, Janet R. Cummings, Xu Ji, Adam S. Wilk

**Affiliations:** 1 Department of Health Policy and Management, Rollins School of Public Health, Emory University, Atlanta, Georgia; 2 Now with Department of Population Health Sciences, Weill Cornell Medicine, New York, New York; 3 Department of Pediatrics, Emory University School of Medicine, Atlanta, Georgia; 4 Children’s Healthcare of Atlanta, Atlanta, Georgia

## Abstract

**Importance:**

Medicaid and Children’s Health Insurance Program cover almost 50% of children with special health care needs (CSHCN). CSHCN often require specialty services and have been increasingly enrolled in Medicaid managed care (MMC) plans, but there is a dearth of recent national studies on specialty care access among publicly insured children and particularly CSHCN.

**Objective:**

To provide recent, nationwide evidence on the association of MMC penetration with specialty care access among publicly insured children, with a special focus on CSHCN.

**Design, Setting, and Participants:**

This cross-sectional study used nationally representative data from the 2016 to 2019 National Survey of Children’s Health to identify publicly insured children in 41 states that administered comprehensive managed care organizations for Medicaid. Data analysis was performed from May 2022 to March 2023.

**Exposure:**

Form CMS-416 data were used to measure state-year level share of Medicaid-enrolled children who were covered by MMC (ie, MMC penetration).

**Main Outcomes and Measures:**

Measures of specialty care access included whether, in the past year, the child had (1) any visit to non–mental health (MH) specialists, (2) any visit to MH professionals, and (3) any unmet health care needs and (4) whether the caregiver ever felt frustrated getting services for their child. Logistic regression models were used to examine the association of MMC penetration with specialty care access among all publicly insured children, and separately for CSHCN and non-CSHCN.

**Results:**

Among 20 029 publicly insured children, 7164 (35.8%) were CSHCN, 9537 (48.2%) were female, 4110 (37.2%) were caregiver-reported Hispanic, and 2812 (21.4%) were caregiver-reported non-Hispanic Black (all percentages are weighted). MMC was not associated with significant changes in any visit to non-MH specialists and unmet health care needs. In addition, MMC penetration was positively associated with caregiver frustration among all children (adjusted odds ratio, 1.23; 95% CI, 1.03-1.48; *P* = .02) and was negatively associated with any visit to MH professionals among CSHCN (adjusted odds ratio, 0.75; 95% CI, 0.58-0.98; *P* = .04).

**Conclusions and Relevance:**

In this cross-sectional study evaluating MMC and specialty care access for publicly insured children, increased MMC enrollment was not associated with improved specialty care access for publicly insured children, including CSHCN. MMC was associated with less access to specialties like MH and increased frustrations among caregivers seeking services for their children.

## Key Points

**Question:**

Is state-level variation in Medicaid managed care (MMC) enrollment among children associated with changes in specialty care access for publicly insured children, especially children with special health care needs (CSHCN)?

**Findings:**

In this cross-sectional study of 20 029 publicly insured children, higher MMC penetration was not associated with non–mental health specialty access and unmet needs, but was associated with greater frustration in care-seeking among caregivers and reduced mental health care access among CSHCN.

**Meaning:**

These findings suggest that MMC enrollment increases may not improve specialty care access for publicly insured children, including CSHCN.

## Teaser

This cross-sectional study examines the association of Medicaid managed care penetration and specialty care access among publicly insured children, with a special focus on children with special health care needs.

## Introduction

Because Medicaid and Children’s Health Insurance Program (CHIP) cover almost half of all children with special health care needs (CSHCN) in the US,^1^ ensuring that publicly insured CSHCN have adequate specialty care access is crucial. The US Maternal and Child Health Bureau defines CSHCN as children who “have or are at increased risk for a chronic physical, developmental, behavioral, or emotional conditions … [and] also require health and related services of a type or amount beyond that required by children generally.”^2,3^ Although CSHCN were historically exempted from Medicaid managed care (MMC), many states have increasingly enrolled CSHCN in MMC in recent years.^4^ As of 2019, CSHCN were mandated to enroll in MMC in 33 states.^5^

Among the states administering MMC, the most common arrangement is to contract with private managed care organizations (MCOs).^6^ Under this arrangement, states pay contracted MCOs capitation payments on a per-enrollee-per-month basis, a payment model that transfers financial risk to the MCOs and encourages them to constrain costs and spending growth.^7^ With these incentives, many MCOs seek to control spending by using cost containment tools, including forming narrow networks of practitioners and institutions and requiring prior authorization for specialty services. Notably, MCOs often focus these cost containment tools on high-cost (eg, specialty) services and on enrollees with high spending or service utilization.^7,8^

CSHCN can experience worse access to specialty care under MMC compared with traditional fee-for-service Medicaid. Because CSHCN usually have higher health service utilization and spending than children who are not CSHCN (hereafter, *non-CSHCN*), they may be more likely to be affected by cost containment tools and encounter barriers to accessing needed specialty services.^9^ Although prior studies^9,10,11,12,13,14,15,16^ showed mixed evidence of MMCs’ impact on specialty care access among children, many of these studies focused on a single state, a restricted geographic region, and/or the expansion of MMC between the mid-1990s and early 2000s. To our knowledge, there is no national evaluation assessing the recent increase of MMC enrollment among children and its impact on specialty care access among publicly insured children and CSHCN.

To address this gap in the literature, we used nationally representative data to provide contemporary evidence on the association between MMC penetration and access to specialty care (including any use of services, perceived unmet need, and perceived difficulties in receiving care) among publicly insured children, with a special focus on CSHCN. Our findings help inform assessments of specialty care access for publicly insured children and CSHCN, as states increasingly enroll them in MMC.

## Methods

### Study Sample

The institutional review board of Emory University determined that this cross-sectional study was not research with human participants or clinical investigation; thus, informed consent was not needed, in accordance with 45 CFR §46. This study followed the Strengthening the Reporting of Observational Studies in Epidemiology (STROBE) reporting guideline for cross-sectional studies.

We derived the study sample from the 2016 to 2019 National Survey of Children’s Health (NSCH). The NSCH is a national survey on children’s health, health service use, and quality of health care received, with a primary goal of assessing the prevalence and impact of special health care needs.^17^ A nationally representative sample of noninstitutionalized children younger than 18 years, including a subsample of CSHCN, is selected through an address-based sampling process. The survey is completed by the child’s primary caregiver via mail-based and web-based instruments. During study years, the weighted survey response rate ranged from 37.4% to 43.1%.^18,19,20,21^

We identified publicly insured children aged 3 to 17 years in 41 states that administered comprehensive risk-based MCOs in Medicaid from 2016 to 2019. Specifically, we excluded 8 states that administered MMC through primary care case management (ie, Alabama, Arkansas [2016-2018], Idaho, Maine, Montana, North Carolina, Oklahoma, and South Dakota) because these plans usually do not apply cost containment tools for specialty care and have been shown to have different impacts on specialty care access than MCOs.^13,15,22,23^ Three states with no MMC were also excluded (ie, Alaska, Connecticut, and Wyoming).

For stratified analyses, we identified CSHCN and non-CSHCN among publicly insured children in the study sample. The NSCH defines CSHCN as children with any medical, behavioral, or other health condition that has lasted or is expected to last 1 year or longer who also meet any of the following criteria: (1) the child needs or uses prescription medications because of the condition, (2) because of the condition the child needs more health services than usual for most children of the same age, (3) the condition limits the child’s ability to do things that most children of the same age can do, (4) the child needs special therapy for the condition, and/or (5) the child needs treatment or counseling for the condition.^24,25,26^ Chronic conditions and disabilities surveyed in NSCH are provided in the eAppendix in Supplement 1.

### MMC Penetration

We derived state-year level MMC penetration among children aged 3 to 18 years from Form CMS-416 data (data for children aged 3 to 17 years were unavailable).^27^ Form CMS-416 provides annual, state-level, age group–specific data on MMC enrollment among Medicaid enrollees.^27^ We estimated MMC penetration across study state-years as the proportion of Medicaid-enrolled children aged 3 to 18 years (ie, *Eligibles for EPSDT* in CMS-416) who were enrolled in MMC (ie, *Eligibles enrolled in managed care* in CMS-416).^28^ We provided MMC penetration data across study state-years in eTable 1 in Supplement 1.

### Outcomes

We examined 4 measures of access to specialty care. Three dichotomous measures assess whether, in the past year, the caregiver reported that the child had (1) any visit to non–mental health (MH) specialists (eg, surgeons or cardiologists), (2) any visit to MH professionals (ie, psychiatrists, psychologists, psychiatric nurses, and clinical social workers), and (3) any unmet health care needs (ie, the primary caregiver reported that the child needed health care but the care was not received, including medical care and MH services). A fourth dichotomous outcome assessed whether the caregiver reported ever feeling frustrated in getting services for their child in the past year.

### Covariates

We drew on the Andersen Behavioral Model for Health Care Utilization and the literature to identify relevant covariates.^29^ At the individual level, predisposing factors included age, sex, and caregiver-reported race and ethnicity. Data on race and ethnicity were included in this study because race and ethnicity are key disposing factors that can influence access to specialty care. Enabling factors included family income and the primary caregiver’s educational attainment. Need-related factors included primary caregiver–rated child health and the number of identified chronic conditions and disabilities the child currently has. At the state level, using state and year identifiers, we linked NSCH data with data from the Area Health Resources Files to include 2 variables that measure state supply of psychiatrists and nonpsychiatrist specialists (eg, cardiologists, anesthesiologists) per 100 000 state population separately.

### Statistical Analysis

Data analysis was performed from May 2022 to March 2023. We estimated logistic regression models to examine the association of state-year–level variation in MMC penetration with each outcome. First, we estimated the association of MMC penetration with each outcome among all publicly insured children. Second, because sample characteristics differ between CSHCN and non-CSHCN, we conducted stratified analysis to examine the association of MMC penetration with each outcome among CSHCN and non-CSHCN separately.

We used a cross-sectional design to examine the association of MMC penetration with access to specialty care. We controlled for covariates and NSCH’s complex survey design in all regression models. In addition, we included survey year to control for confounding effects of national year-varying trends (eg, national economy) and state fixed effects (ie, series of state-identifying dummy variables) to control for time-invariant differences across states (eg, the state Medicaid office’s contracting approach). Observations with missing values were excluded from analyses. See the sample derivation flowchart in the eFigure in Supplement 1 and study sample size by state-year in eTable 2 in Supplement 1. Data were analyzed using Stata statistical software version 17 SE (StataCorp LLC). We used the margins command to calculate estimated probabilities and the associated 95% CIs. Two-sided *P* < .05 was considered statistically significant. We applied the svy and subpop commands to calculate estimates for our analytics sample and used the full data in NSCH to generate robust SEs (ie, Huber-White sandwich estimators) that properly adjusted for NSCH’s complex survey design.^21,30^

For ease of interpretation, we standardized state-year–level MMC penetration (mean [SD], 89.8% [6.0%]) for analysis. Results of regression models were interpreted as the change in outcome associated with a 1 SD increase (ie, 6.0 percentage points) in MMC penetration.

## Results

### Sample Characteristics

Among 20 029 publicly insured children (weighted sample size, 18 618 645 children), 7164 (35.8%) were CSHCN, 6773 (33.8%) were 10 to 14 years old, 9537 (48.2%) were female, 4110 (37.2%) were caregiver-reported Hispanic, and 2812 (21.4%) were caregiver-reported non-Hispanic Black (all percentages for children throughout the Results section are weighted). Compared with their peers who were non-CSHCN (12 865 children), the CSHCN were more likely to visit non-MH specialists (2526 children [33.0%] vs 1044 children [7.0%]) and MH professionals (2846 children [36.1%] vs 668 children [4.0%]). Moreover, CSHCN were more likely than their peers to have unmet health care needs (579 children [10.3%] vs 328 children [2.4%]). Caregivers of CSHCN were also more than twice as likely to report feeling frustrated in getting services for their child (2843 children [42.9%] vs 2024 children [15.9%]) (Table 1).

**Table 1.** Sample Characteristics of Publicly Insured Children in Study States, National Survey of Children’s Health, 2016-2019

Characteristic	Publicly insured children, unweighted No. (weighted %)			*P* value
	Total	Non-CSHCN	CSHCN	
Sample size, No. of children				
Unweighted	20 029	12 865	7164	NA
Weighted	18 618 645	13 440 947	5 177 698	NA
Outcomes				
Any visit to non–mental health specialists	3570 (14.2)	1044 (7.0)	2526 (33.0)	<.001
Any visit to mental health professionals	3514 (12.9)	668 (4.0)	2846 (36.1)	<.001
Any reported unmet health care needs	907 (4.6)	328 (2.4)	579 (10.3)	<.001
Caregiver ever felt frustrated in getting service for the child	4867 (23.4)	2024 (15.9)	2843 (42.9)	<.001
Covariates				
Predisposing factors				
Age group, y				
3-5	3816 (20.5)	2939 (23.1)	877 (13.7)	<.001
6-9	4952 (28.0)	3180 (27.5)	1772 (29.3)	
10-14	6773 (33.8)	4043 (32.5)	2730 (37.2)	
15-17	4488 (17.8)	2703 (16.9)	1785 (19.9)	
Sex				
Female	9537 (48.2)	6507 (50.6)	3030 (41.8)	<.001
Male	10 492 (51.8)	6358 (49.4)	4134 (58.2)	
Caregiver-reported race and ethnicity of the child				
Hispanic	4110 (37.2)	2925 (40.1)	1185 (29.7)	<.001
Non-Hispanic Asian, American Indian, Alaska Native, Native Hawaiian, or Other Pacific Islander	1046 (4.0)	843 (4.7)	203 (2.0)	
Non-Hispanic Black	2812 (21.4)	1787 (20.5)	1025 (23.9)	
Non-Hispanic White	10 486 (32.5)	6346 (30.0)	4140 (38.9)	
Other^a^	1575 (4.9)	964 (4.7)	611 (5.5)	
Enabling factors				
Family income, % of Federal Poverty Level				
<100	6495 (42.2)	4182 (41.6)	2313 (43.9)	.002
100-200	7281 (36.8)	4912 (38.0)	2369 (33.7)	
>200-300	3290 (12.2)	2109 (12.3)	1181 (11.9)	
>300	2963 (8.8)	1662 (8.1)	1301 (10.4)	
Primary caregiver’s education attainment				
Less than high school	1328 (17.8)	955 (19.4)	373 (13.8)	<.001
High school	5754 (34.1)	3861 (34.5)	1893 (33.0)	
Some college	7152 (28.7)	4504 (27.8)	2648 (31.0)	
College or higher	5545 (19.4)	3381 (18.4)	2164 (22.2)	
Need-related factors				
Caregiver-rated fair or poor health (vs good, very good, or excellent)	655 (3.1)	70 (0.7)	585 (9.3)	<.001
No. of the child’s chronic conditions or disabilities^b^				
0	9231 (53.4)	8830 (71.7)	401 (5.7)	<.001
1	3924 (19.0)	2681 (19.8)	1243 (16.9)	
2-3	3664 (15.4)	1188 (7.3)	2476 (36.5)	
4-17	3210 (12.2)	166 (1.2)	3044 (41.0)	
Contextual factors				
State-level supply of nonpsychiatrist specialists, mean (SD), No./100 000 population	168.1 (0.5)	168.5 (0.6)	167.1 (0.8)	.20
State-level supply of psychiatrists, mean (SD), No./100 000 population	11.7 (0.1)	11.8 (0.1)	11.4 (0.1)	.007

Abbreviations: CSHCN, Children with Special Health Care Needs; NA, not applicable.

^a^ Other race and ethnicity included children who were identified as non-Hispanic other race not listed or 2 or more races by their primary caregiver.

^b^ The list of chronic conditions and disabilities surveyed in National Survey of Children’s Health is shown in the eAppendix in Supplement 1.

Many sample characteristics differed significantly between CSHCN and non-CSHCN. CSHCN were more likely to than their peers to be older (10-14 years old, 2730 children [37.2%] vs 4043 children [32.5%]), male (4134 children [58.2%] vs 6358 children [49.4%]), and caregiver-reported non-Hispanic White (4140 children [38.9%] vs 6346 children [30.0%]). CSHCN were also more likely than their peers to have a family income less than 100% of the Federal Poverty Level (2313 children [43.9%] vs 4182 children [41.6%]) and to have 1 or more caregiver-reported identified chronic conditions and disabilities (no identified condition or disability, 401 children [5.7%] vs 8830 children [71.7%]) (Table 1).

### Association of MMC Penetration With Specialty Care Access

Among all publicly insured children, we found no statistically significant association of MMC penetration with any visit to non-MH specialists (adjusted odds ratio [aOR], 0.93; 95% CI, 0.74-1.15; *P* = .48), any visit to MH professionals (aOR, 0.85; 95% CI, 0.69-1.05; *P* = .13), and any unmet health care needs (aOR, 0.94; 95% CI, 0.68-1.30; *P* = .73) (Table 2). In contrast, MMC penetration was positively associated with caregiver frustration in getting services for their child (aOR, 1.23; 95% CI, 1.03-1.48; *P* = .02) (Table 2). For example, a 1 SD (ie, 6 percentage points) increase above the mean MMC penetration was associated with an increase in the estimated probability of caregiver frustration from 19.5% (95% CI, 17.9%-21.1%) to 23.0% (95% CI, 19.9%-26.2%) (Table 3).

**Table 2.** Logistic Regression Models Examining the Association of MMC Penetration With Outcomes Among All Publicly Insured Children^a^

Variable	aOR (95% CI)			
	Any visit to non-MH specialists	Any visit to MH professionals	Any unmet health care needs	Ever felt frustrated in getting service for the child
Sample size, No. of children				
Unweighted	19 563	19 625	19 634	19 599
Weighted	18 222 557	18 284 271	18 279 117	18 290 437
MMC penetration (standardized)	0.93 (0.74-1.15)	0.85 (0.69-1.05)	0.94 (0.68-1.30)	1.23 (1.03-1.48)
Covariates				
Predisposing factors				
Age, y (reference, 3-5 y)				
6-9	0.82 (0.64-1.05)	3.11 (2.22-4.37)	1.21 (0.77-1.91)	1.01 (0.81-1.26)
10-14	0.96 (0.75-1.22)	4.19 (3.06-5.74)	1.34 (0.88-2.04)	0.96 (0.78-1.12)
15-17	1.13 (0.88-1.46)	5.12 (3.65-7.18)	2.44 (1.54-3.86)	1.20 (0.94-1.52)
Female sex	1.02 (0.86-1.20)	1.06 (0.89-1.27)	1.40 (1.06-1.83)	1.04 (0.89-1.20)
Race and ethnicity (reference, Hispanic)				
Non-Hispanic Asian, American Indian, Alaska Native, Native Hawaiian and Other Pacific Islander	0.70 (0.46-1.07)	0.85 (0.53-1.37)	0.77 (0.40-1.47)	1.02 (0.74-1.42)
Non-Hispanic Black	0.65 (0.50-0.86)	0.83 (0.62-1.11)	1.58 (0.95-2.62)	1.03 (0.80-1.33)
Non-Hispanic White	1.12 (0.90-1.41)	1.19 (0.94-1.50)	1.24 (0.86-1.79)	1.20 (1.00-1.45)
Other^b^	1.29 (0.86-1.95)	0.97 (0.69-1.37)	1.39 (0.86-2.24)	1.52 (1.13-2.06)
Enabling factors				
Primary caregiver’s education attainment (reference, less than high school)				
High school	1.06 (0.75-1.50)	0.98 (0.69-1.40)	1.08 (0.64-1.82)	0.95 (0.71-1.26)
Some college	1.21 (0.85-1.71)	1.17 (0.82-1.67)	1.38 (0.85-2.24)	1.10 (0.83-1.47)
College or higher	1.75 (1.22-2.50)	1.12 (0.78-1.60)	1.42 (0.85-2.40)	1.25 (0.93-1.68)
Family income, % of Federal Poverty Level (reference, <100%)				
100-200	1.13 (0.92-1.38)	1.06 (0.85-1.33)	0.93 (0.67-1.28)	0.95 (0.80-1.14)
>200-300	1.21 (0.88-1.67)	1.11 (0.83-1.49)	0.63 (0.42-0.94)	0.89 (0.70-1.13)
>300	1.39 (1.09-1.76)	1.49 (1.15-1.93)	0.48 (0.32-0.72)	0.87 (0.69-1.10)
Need-related factors				
Parent-rated fair or poor health (vs good, very good, or excellent)	2.38 (1.67-3.40)	0.86 (0.60-1.23)	1.20 (0.72-2.01)	1.78 (1.20-2.65)
No. of chronic conditions or disabilities (reference, 0)^c^				
1	3.39 (2.60-4.43)	3.03 (2.21-4.41)	1.92 (1.32-2.79)	1.71 (1.39-2.10)
2-3	5.91 (4.66-7.50)	8.68 (6.54-11.52)	3.44 (2.18-5.42)	2.80 (2.28-3.43)
4-17	10.90 (8.54-13.92)	33.37 (25.00-44.55)	7.66 (5.17-11.33)	7.14 (5.75-8.88)
Contextual factors				
State-level supply of nonpsychiatrist specialists, No./100 000 population	0.98 (0.96-1.00)	1.01 (0.99-1.03)	1.02 (0.99-1.05)	1.02 (1.00-1.04)
State-level supply of psychiatrists, No./100 000 population	0.84 (0.35-1.99)	0.99 (0.37-2.69)	0.58 (0.14-2.32)	1.72 (0.80-3.69)

Abbreviations: aOR, adjusted odds ratio; MH, mental health; MMC, Medicaid managed care.

^a^ Differences in sample size across models are due to exclusion of missing data. For ease of interpretation, we standardized state-year–level MMC penetration for analysis. Results of regression models were interpreted as estimates associated with a 1 SD increase in the level of MMC penetration (ie, 6.0 percentage points).

^b^ Other race and ethnicity included children who were identified as non-Hispanic other race not listed or 2 or more races by their primary caregiver.

^c^ The list of chronic conditions and disabilities surveyed in National Survey of Children’s Health is shown in the eAppendix in Supplement 1.

**Table 3.** Estimated Probabilities of Outcomes Associated With MMC Penetration Among Publicly Insured Children^a^

MMC penetration	Estimated probability, % (95% CI)			
	Any visit to non-MH specialists	Any visit to MH professionals	Any unmet health care needs	Ever felt frustrated in getting service for the child
Among all publicly insured children				
6 Percentage point decrease	12.5 (9.4-15.6)	8.0 (5.8-10.1)	2.4 (1.3-3.5)	16.4 (13.2-19.7)
Mean (89.8%)	11.6 (10.4-12.9)	6.9 (5.8-7.9)	2.3 (1.7-2.8)	19.5 (17.9-21.1)
6 Percentage point increase	10.9 (8.8-12.9)	5.9 (4.6-7.2)	2.2 (1.5-2.8)	23.0 (19.9-26.2)
Among publicly insured non-CSHCN				
6 Percentage point decrease	8.6 (4.7-12.5)	3.7 (1.6-5.9)	1.4 (0.6-2.2)	14.8 (11.3-18.2)
Mean (89.8%)	8.2 (6.9-9.5)	4.1 (3.2-5.1)	1.7 (1.1-2.3)	17.9 (15.9-19.9)
6 Percentage point increase	7.8 (5.3-10.4)	4.6 (2.7-6.4)	2.1 (1.2-3.1)	21.4 (17.5-25.4)
Among publicly insured CSHCN				
6 Percentage point decrease	31.8 (23.3-40.4)	27.4 (18.2-36.7)	6.3 (1.2-11.4)	32.0 (23.7-40.3)
Mean (89.8%)	29.7 (24.1-35.4)	22.2 (16.6-27.7)	5.1 (2.4-7.8)	35.4 (29.5-41.3)
6 Percentage point increase	27.7 (20.5-35.0)	17.7 (12.4-23.0)	4.1 (1.9-6.3)	39.0 (31.3-46.6)

Abbreviations: CSHCN, Children with Special Health Care Needs; MH, Mental Health; MMC, Medicaid managed care.

^a^ For ease of interpretation, MMC penetration was standardized for analysis (mean [SD], 89.8% [6.0%]) and 1 SD increase was approximately a 6–percentage point higher level of MMC penetration. We report the estimated probability of each outcome at different value of MMC penetration while holding all other covariates at their means by using the margins and the atmeans command in Stata statistical software. All logistic regression models control for predisposing factors (ie, age, sex, and caregiver-reported race and ethnicity), enabling factors (ie, family income and the primary caregiver’s educational attainment), need-related factors (ie, primary caregiver–rated child health and number of identified chronic condition or disabilities), state supply of nonpsychiatrist specialists, state supply of psychiatrists, sampling weights, survey year (ie, year-fixed effects), and state identifiers (ie, state-fixed effects). Sample size differences were the result of exclusion of missing values for analyses.

In stratified analyses for CSHCN and non-CSHCN, we found that MMC penetration was not associated with significant changes in outcomes with 2 exceptions. First, among CSHCN, MMC penetration was negatively associated with having any visit to MH professionals (aOR, 0.75; 95% CI, 0.58-0.98; *P* = .04) (Table 4). Specifically, a 6–percentage point increase above the mean MMC penetration was associated with a decrease in the estimated probability of having any visit to MH professionals from 22.2% (95% CI, 16.6%-27.7%) to 17.7% (95% CI, 12.4%-23.0%) (Table 3). Second, the aOR for MMC penetration was greater than 1.00 for caregiver frustration in both subsamples, although it was statistically significant only for non-CSHCN (aOR, 1.28; 95% CI, 1.01-1.62; *P* = .04) (Table 5).

**Table 4.** Stratified Analyses of the Association of MMC Penetration With Any Use of Specialty Care Among Non-CSHCN and CSHCN^a^

Variable	aOR (95% CI)			
	Any visit to non-MH specialists		Any visit to MH professionals	
	Non-CSHCN	CSHCN	Non-CSHCN	CSHCN
Sample size, No. of children				
Unweighted	12 560	7003	12 602	7023
Weighted	13 156 942	5 065 615	13 211 419	5 072 852
MMC penetration (standardized)	0.95 (0.64-1.42)	0.91 (0.70-1.18)	1.11 (0.70-1.75)	0.75 (0.58-0.98)
Covariates				
Predisposing factors				
Age, y (reference, 3-5 y)				
6-9	1.01 (0.67-1.53)	0.59 (0.42-0.83)	3.79 (2.08-6.90)	2.87 (1.89-4.38)
10-14	1.29 (0.87-1.91)	0.68 (0.49-0.95)	5.12 (2.91-9.03)	4.02 (2.71-5.95)
15-17	1.69 (1.13-2.52)	0.75 (0.53-1.06)	6.47 (3.52-11.90)	5.28 (3.50-7.97)
Female sex	1.06 (0.80-1.40)	1.06 (0.86-1.30)	1.16 (0.83-1.62)	1.04 (0.84-1.28)
Race and ethnicity (reference, Hispanic)				
Non-Hispanic Asian, American Indian, Alaska Native, Native Hawaiian, or Other Pacific Islander	0.66 (0.32-1.32)	0.80 (0.46-1.41)	0.32 (0.13-0.80)	1.64 (0.90-2.99)
Non-Hispanic Black	0.63 (0.41-0.97)	0.67 (0.47-0.94)	0.61 (0.37-1.01)	0.90 (0.62-1.31)
Non-Hispanic White	1.35 (0.96-1.92)	1.02 (0.76-1.36)	1.13 (0.78-1.64)	1.22 (0.91-1.64)
Other^b^	1.63 (0.80-3.32)	1.08 (0.72-1.63)	0.85 (0.48-1.50)	1.05 (0.70-1.59)
Enabling factors				
Primary caregiver’s education attainment (reference, less than high school)				
High school	0.89 (0.53-1.52)	1.15 (0.75-1.77)	1.05 (0.54-2.04)	0.85 (0.56-1.29)
Some college	1.01 (0.59-1.71)	1.28 (0.82-2.02)	1.14 (0.59-2.20)	1.07 (0.70-1.62)
College or higher	1.49 (0.87-2.56)	1.74 (1.10-2.76)	1.16 (0.60-2.26)	0.90 (0.59-1.38)
Family income, % of Federal Poverty Level (reference, <100%)				
100-200	1.09 (0.79-1.50)	1.24 (0.94-1.64)	1.35 (0.88-2.05)	0.98 (0.75-1.29)
>200-300	1.22 (0.70-2.14)	1.23 (0.89-1.69)	1.36 (0.79-2.34)	1.00 (0.71-1.42)
>300	1.04 (0.70-1.55)	1.67 (1.22-2.30)	1.62 (1.03-2.56)	1.49 (1.09-2.04)
Need-related factors				
Parent-rated fair or poor health (vs good, very good, or excellent)	0.71 (0.25-2.06)	2.32 (1.57-3.44)	0.54 (0.09-3.22)	0.75 (0.52-1.08)
No. of chronic conditions or disabilities (reference, 0)^c^				
1	3.11 (2.20-4.40)	1.00 (0.61-1.63)	2.61 (1.71-3.99)	0.85 (0.48-1.51)
2-3	3.09 (2.19-4.36)	1.51 (0.94-2.42)	3.67 (2.51-5.36)	1.79 (1.04-3.09)
4-17	7.83 (3.73-16.44)	2.15 (1.35-3.40)	15.12 (7.27-31.45)	5.21 (3.06-8.89)
Contextual factors				
State-level supply of nonpsychiatrist specialists, No./100 000 population	0.97 (0.94-1.01)	0.98 (0.96-1.00)	1.03 (0.99-1.07)	1.00 (0.98-1.03)
State-level supply of psychiatrists. No./100 000 population	0.89 (0.20-4.02)	0.70 (0.24-2.02)	2.78 (0.44-17.45)	0.51 (0.15-1.68)

Abbreviations: aOR, adjusted odds ratio; CSHCN, children with special health care needs; MH, mental health; MMC, Medicaid managed care.

^a^ Differences in sample size across models are due to exclusion of missing data. For ease of interpretation, we standardized state-year–level MMC penetration for analysis. Results of regression models were interpreted as estimates associated with a 1 SD increase in the level of MMC penetration (ie, 6.0 percentage points).

^b^ Other race and ethnicity included children who were identified as non-Hispanic other race not listed or 2 or more races by their primary caregiver.

^c^ The list of chronic conditions and disabilities surveyed in National Survey of Children’s Health is shown in eAppendix 1 in Supplement 1.

**Table 5.** Stratified Analyses of the Association of MMC Penetration With Any Unmet Health Care Needs and Caregiver Ever Felt Frustrated in Efforts to Get Service Among Non-CSHCN and CSHCN^a^

Variable	aOR (95% CI)			
	Any unmet health care needs		Ever felt frustrated in getting service for the child	
	Non-CSHCN	CSHCN	Non-CSHCN	CSHCN
Sample size, No. of children				
Unweighted	12 360	7025	12 587	7012
Weighted	13 153 058	5 093 706	13 197 635	5 092 802
MMC penetration (standardized)	1.23 (0.82-1.83)	0.80 (0.51-1.27)	1.28 (1.01-1.62)	1.18 (0.91-1.52)
Covariates				
Predisposing factors				
Age, y (reference, 3-5)				
6-9	1.10 (0.62-1.94)	1.20 (0.62-2.33)	1.06 (0.79-1.43)	0.82 (0.59-1.14)
10-14	1.36 (0.77-2.42)	1.32 (0.71-2.44)	1.11 (0.84-1.46)	0.70 (0.51-0.97)
15-17	2.84 (1.54-5.24)	2.26 (1.16-4.44)	1.42 (1.04-1.94)	0.86 (0.60-1.24)
Female sex	1.22 (0.82-1.83)	1.55 (1.09-2.22)	1.10 (0.89-1.35)	0.99 (0.80-1.23)
Race and ethnicity (reference, Hispanic)				
Non-Hispanic Asian, American Indian, Alaska Native, Native Hawaiian, or Other Pacific Islander	0.81 (0.31-2.07)	0.73 (0.29-1.87)	0.93 (0.63-1.37)	1.25 (0.66-2.34)
Non-Hispanic Black	1.66 (0.84-3.28)	1.42 (0.75-2.66)	0.90 (0.66-1.22)	1.32 (0.90-1.93)
Non-Hispanic White	1.67 (0.91-3.06)	0.93 (0.60-1.45)	1.06 (0.83-1.35)	1.48 (1.11-1.98)
Other^b^	1.97 (0.93-4.16)	1.06 (0.58-1.94)	1.77 (1.18-2.64)	1.31 (0.89-1.92)
Enabling factors				
Primary caregiver’s education attainment (reference, less than high school)				
High school	1.07 (0.49-2.36)	1.04 (0.50-2.17)	0.84 (0.59-1.21)	1.14 (0.73-1.78)
Some college	1.18 (0.57-2.44)	1.47 (0.74-2.94)	1.10 (0.76-1.58)	1.14 (0.73-1.78)
College or higher	1.30 (0.62-2.71)	1.43 (0.67-3.08)	1.05 (0.72-1.52)	1.52 (0.96-2.40)
Family income, % of Federal Poverty Level (reference, <100%)				
100-200	0.96 (0.63-1.46)	0.96 (0.62-1.50)	0.90 (0.71-1.13)	1.09 (0.83-1.42)
>200-300	0.57 (0.32-1.03)	0.68 (0.39-1.18)	0.89 (0.64-1.26)	0.85 (0.62-1.18)
>300	0.42 (0.21-0.84)	0.52 (0.30-0.90)	0.79 (0.56-1.11)	0.99 (0.72-1.35)
Need-related factors				
Parent-rated fair or poor health (vs good, very good, or excellent)	3.46 (0.89-13.51)	0.96 (0.55-1.67)	4.23 (2.14-8.37)	1.33 (0.88-2.01)
No. of chronic conditions or disabilities (reference, 0)^c^				
1	1.55 (1.01-2.38)	1.14 (0.43-2.99)	1.47 (1.16-1.88)	1.01 (0.61-1.66)
2-3	1.98 (0.96-4.10)	1.28 (0.49-3.36)	1.90 (1.37-2.64)	1.25 (0.78-2.01)
4-17	6.69 (2.62-17.09)	2.54 (1.02-6.35)	4.16 (2.28-7.59)	2.94 (1.84-4.71)
Contextual factors				
State-level supply of nonpsychiatrist specialists, No./100 000 population	1.04 (1.00-1.09)	1.00 (0.96-1.03)	1.01 (0.99-1.04)	1.03 (1.01-1.05)
State-level supply of psychiatrists. No./100 000 population	3.72 (0.41-33.51)	0.16 (0.03-0.90)	2.83 (0.93-8.60)	0.96 (0.35-2.64)

Abbreviations: aOR, adjusted odds ratio; CSHCN, children with special health care needs; MMC, Medicaid managed care.

^a^ Differences in sample size across models are due to exclusion of missing data. For ease of interpretation, we standardized state-year–level MMC penetration for analysis. Results of regression models were interpreted as estimates associated with a 1 SD increase in the level of MMC penetration (ie, 6.0 percentage points).

^b^ Other race and ethnicity included children who were identified as non-Hispanic other race not listed or 2 or more races by their primary caregiver.

^c^ The list of chronic conditions and disabilities surveyed in National Survey of Children’s Health is shown in eAppendix 1 in Supplement 1.

## Discussion

Considering recent increases in MMC enrollment among children, in this cross-sectional study we provide contemporary evidence for the association of MMC penetration with access to specialty care among publicly insured children, overall and by special health care needs status. We found that MMC was not associated with significant changes in any use of non-MH specialty care or unmet health care needs. However, increased MMC penetration was associated with increased caregivers’ frustration in getting services for their publicly insured child and a lower probability of visiting MH professionals among CSHCN. Our findings indicate that increasing MMC enrollment may have limited impact on changing specialty care access for publicly insured children, including CSHCN, with the few changes limited to select specialties like MH and caregivers’ frustrations in seeking care.

We found no significant association between MMC and any use of non-MH specialty care or unmet health care needs among publicly insured children, including CSHCN. Many state-level evaluations have indicated that children, including CSHCN, in MMC experienced a similar level of access to non-MH specialty care and reported similar rates of unmet health care needs compared with their counterparts in fee-for-service Medicaid or CHIP.^11,12,31,32,33^ Although the broader evidence on this association has been mixed,^11,12,31,32,33,34,35,36^ our contemporary nationwide findings align with these state-level studies, supporting the absence of an association. Our findings suggest that access to non-MH specialty care and unmet health care needs may not worsen overall as states increase MMC enrollment among children.

Several potential reasons may explain our finding of a positive association between MMC penetration and caregivers’ frustration in getting services for their child. First, caregivers often perceive inadequate practitioner availability within MMC plans.^37^ Although enrollees commonly rely on MMC plans’ practitioner directories to identify in-network practitioners, these directories are often inaccurate and overstate practitioners’ availability, especially for specialists.^38,39,40,41^ Previous studies^38,40^ found that more than 50% of practitioners listed in these directories did not offer an appointment for new patients. Moreover, specialty care is often highly concentrated in MMC networks of practitioners and institutions with a small group of core practitioners, who provide most specialty care.^39^ Caregivers may encounter challenges in identifying these core practitioners or face difficulties in accessing them because of long travel distances. Finally, MMC plans experience high physician turnover. On average, more than 30% of primary care physicians leave an MMC plan network within 4 years,^42^ and MCOs have reported greater difficulty in retaining specialists than primary care physicians.^43^ When physicians leave MMC plans, caregivers of children receiving ongoing treatment may struggle to identify acceptable in-network replacement practitioners, resulting in increased frustration.

We also found that MMC penetration was negatively associated with having any visit to MH professionals among CSHCN, but not among non-CSHCN. This negative association is consistent with previous studies among Medicaid-enrolled children^10^; however, extant evidence on MMC’s impact on MH care access among CSHCN has been mixed.^12^ Many CSHCN have multiple conditions and disabilities requiring frequent visits to various specialists.^44^ Consequently, they may be more likely to experience barriers to accessing MH services under MMC, in part, because many CSHCN require MH treatment from specialty MH practitioners; however, the availability of specialty MH practitioners remains low in MMC.^41,45,46,47^ In addition, because many states allow MCOs to carve out MH services (meaning that MH benefits may be administered by separate behavioral health MMC plans), many publicly insured children can experience additional administrative burden coordinating with multiple MMC plans when seeking MH care under MMC. CSHCN enrolled in multiple MMC plans may be more likely to experience these burdens if they require regular MH care from specialist practitioners.

Despite few associations between MMC enrollment and access to specialty services, limited MH care access, and caregiver frustration, it is important to recognize that many publicly insured children and CSHCN have difficulty accessing specialty care regardless of MMC enrollment. Several interventions may facilitate specialty care access for children enrolled in both MMC and fee-for-service Medicaid. A notable concern within Medicaid (including MMC) is low specialist participation, partly related to low reimbursement rates, administrative complexities in billing, delayed reimbursements, and frequent claims denials.^41,43,48,49,50^ To improve specialist participation, states may consider increasing reimbursement rates, simplifying billing procedures, expediting the reimbursement process, and improving administrative protocols to address claims denials and resubmissions.^41,49,50^ Moreover, practitioner shortages, especially among specialists and pediatric subspecialists, can further limit specialty care access for publicly insured children.^51,52,53,54^ Such challenges may be ameliorated by expanding the use of telehealth for patient care, case management, and case discussions between specialists and primary care practitioners.^55^

Our findings also offer insights into interventions that may ensure adequate specialty care access for children enrolled in MMC. First, to reduce caregivers’ frustration in identifying practitioners for their child, states can improve the accuracy of MMC plans’ practitioner directories by conducting direct tests (eg, making phone calls to practitioners) and using claims data to identify practitioners who are not actively treating MMC enrollees.^39,56^ Second, states may also consider administering special needs plans designed to provide needed care for different populations of publicly insured children. For example, to better meet the needs of CSHCN with MH conditions, states can provide incentives to special needs plans serving these beneficiaries (eg, paying higher capitation payments) and require the plans to include practitioners who are more experienced in coordinating MH care in their networks of practitioners and institutions.

### Limitations

This study has some limitations. First, we used repeated cross-sectional data from the NSCH; therefore, causality could not be established. Similarly, this study is subject to general limitations of survey data, including recall bias, nonresponse bias, and bias related to caregiver proxy reporting. Second, Form CMS-416 data do not include CHIP-enrolled children. Although all study states (except Washington state) administered their CHIP programs through CHIP Medicaid expansion programs with the same benefits for Medicaid and CHIP enrollees,^57^ the absence of data on managed care penetration among CHIP-enrolled children obscures whether our measure of MMC penetration may overestimate or underestimate managed care penetration among all publicly insured children. Third, for stratified analyses, data were not available to identify MMC penetration among CSHCN and among non-CSHCN separately. We expect the MMC penetration among CSHCN would be lower than the MMC penetration among all children.^58^ If we overestimated MMC penetration among CSHCN, the observed associations between MMC penetration and each outcome among CSHCN would be biased toward the null. Fourth, some measures of specialty care access (eg, unmet needs for different specialty services or number of specialty care visits) that could be impacted by MMC were not examined, an area meriting future research. Specifically, previous studies^9,59^ have suggested that MMCs’ impact on specialty care access may differ by specialties. Null results of this study may reflect heterogeneous treatment effects of MMC on access to different specialty services. Fifth, our models may not capture some state variations in MMC plans; this is an area that warrants future research, including studies using different analytic approaches (eg, multilevel modeling) or data (eg, claims) and incorporating other state-level information on MMC (eg, mandatory vs voluntary enrollment in MMC of select eligibility groups).

## Conclusions

Using nationally representative samples, we provide recent evidence on the association of MMC penetration with access to specialty care among publicly insured children, including CSHCN. We found no significant association of MMC with access to non-MH specialty care or unmet health care needs. The few changes significantly associated with increased MMC penetration included increased frustration among caregivers in getting services for their publicly insured child and decreased access to MH care among CSHCN. As states increasingly enroll children with more complex needs under MMC, it remains important to monitor the landscape of specialty care access, particularly among CSHCN, and to undertake any additional efforts needed to ensure adequate specialty care access for publicly insured children.

## Notes

Supplement 1. **eAppendix.** Chronic Conditions and Disabilities Identified in the National Survey of Children’s Health **eTable 1.** Medicaid Managed Care Penetration Among Children Aged 3-18 Across Study State-Years **eFigure.** Sample Derivation Flowchart **eTable 2.** Weighted and Unweighted Sample Size Across Study State-Years, 2016-2019

Supplement 2.
Data Sharing Statement
