# Supplementary material for: State-Level Variation in Medicaid Managed Care Enrollment and Specialty Care for Publicly Insured Children
Source: JAMA Netw Open. 2023 Oct 5;6(10):e2336415. doi: 10.1001/jamanetworkopen.2023.36415 (PMC10556966; doi:10.1001/jamanetworkopen.2023.36415)
Supplement: Supplement 1. — eAppendix. Chronic Conditions and Disabilities Identified in the National Survey of Children’s Health eTable 1. Medicaid Managed Care Penetration Among Children Aged 3-18 Across Study State-Years eFigure. Sample Derivation Flowchart eTable 2. Weighted and Unweighted Sample Size Across Study State-Years, 2016-2019 [file jamanetwopen-e2336415-s001.pdf]

Supplemental Online Content

Hu JC, Cummings JR, Ji X, Wilk AS. State-level variation in Medicaid managed care enrollment and specialty care for publicly insured children. *JAMA Netw Open*. 2023;6(10):e2336415. doi:10.1001/jamanetworkopen.2023.36415

eAppendix. Chronic Conditions and Disabilities Identified in the National Survey of Children's Health

eTable 1. Medicaid Managed Care Penetration Among Children Aged 3-18 Across Study State-Years

eFigure. Sample Derivation Flowchart

eTable 2. Weighted and Unweighted Sample Size Across Study State-Years, 2016-2019

This supplemental material has been provided by the authors to give readers additional information about their work.

© 2023 Hu JC et al. *JAMA Network Open.*

eAppendix. Chronic Conditions and Disabilities Identified in the National Survey of Children's Health

1. Allergies
2. Arthritis
3. Asthma
4. Brain/head injury or concussion
5. Cerebral Palsy
6. Diabetes
7. Epilepsy or Seizure Disorder
8. Heart condition
9. Frequent or severe headaches or migraine
10. Tourette Syndrome
11. Anxiety problems
12. Depression
13. Substance Use Disorder
14. Down Syndrome
15. Blood Disorders
16. Cystic Fibrosis
17. Other genetic/inherited condition
18. Autism
19. ADD or ADHD
20. Behavioral or conduct problems
21. Developmental Delay
22. Intellectual Disability
23. Speech or other language disorder
24. Learning Disability
25. Blindness or problems with seeing, even when wearing glasses
26. Deafness or problems with hearing

Source: Health Resources and Services Administration. Full-Length NSCH Survey Instruments. Accessed May 13, 2022. https://www.childhealthdata.org/learn-about-the-nsch/survey-instruments

© 2023 Hu JC et al. *JAMA Network Open.*

eTable 1. Medicaid Managed Care Penetration Among Children Aged 3-18 Across Study State-Years

	Medicaid managed care penetration among children aged 3-18			
**State**	**2016**	**2017**	**2018**	**2019**
Arizona	96.6%	90.9%	90.3%	90.4%
Arkansas[a]	N/A	N/A	N/A	92.8%
California	91.8%	91.0%	90.9%	90.6%
Colorado	95.9%	95.5%	92.2%	93.4%
Delaware	95.2%	91.6%	94.8%	94.9%
District of Columbia	91.1%	89.8%	91.2%	91.1%
Florida	93.5%	94.5%	93.8%	93.8%
Georgia	91.3%	91.2%	92.2%	92.1%
Hawaii	85.8%	90.8%	92.4%	90.9%
Illinois	86.6%	85.7%	86.6%	85.0%
Indiana	85.5%	90.8%	87.2%	86.4%
Iowa	90.5%	92.5%	92.4%	93.0%
Kansas	97.5%	95.0%	96.6%	95.9%
Kentucky	96.4%	96.3%	96.4%	96.6%
Louisiana	98.0%	98.4%	97.8%	98.4%
Maryland	94.5%	96.6%	96.6%	96.8%
Massachusetts	78.3%	78.4%	81.6%	80.4%
Michigan	84.1%	85.1%	84.7%	84.4%
Minnesota	82.5%	82.7%	83.2%	84.2%
Mississippi	91.8%	92.1%	92.0%	91.6%
Missouri	67.2%	92.7%	91.6%	91.7%
Nebraska	94.0%	95.4%	95.9%	95.5%
Nevada	76.8%	71.8%	72.6%	72.3%
New Hampshire	92.9%	92.4%	92.2%	93.2%
New Jersey	94.4%	94.9%	95.0%	93.9%
New Mexico	86.3%	87.6%	87.2%	88.3%
New York	93.5%	93.3%	91.6%	91.4%
North Dakota	79.7%	80.1%	76.0%	82.1%
Ohio	90.1%	92.7%	94.0%	93.7%
Oregon	88.2%	88.1%	89.6%	89.7%
Pennsylvania	90.5%	93.1%	93.3%	93.0%
Rhode Island	88.4%	89.7%	90.0%	90.3%
South Carolina	83.8%	86.9%	87.6%	87.6%
Tennessee	98.1%	97.0%	97.1%	96.2%
Texas	86.3%	94.7%	95.1%	94.6%
Utah	79.2%	78.0%	78.2%	78.4%

© 2023 Hu JC et al. *JAMA Network Open.*

Vermont	80.3%	81.2%	80.4%	77.4%
Virginia	89.4%	89.9%	93.1%	94.7%
Washington	90.5%	93.3%	92.9%	92.7%
West Virginia	74.2%	88.8%	88.0%	86.8%
Wisconsin	85.3%	83.7%	84.1%	83.7%

Note: We included states administered Medicaid program through comprehensive risk-based managed care organizations from 2016 to 2019 for analysis. [a]Arkansas switched its Medicaid program from primary care case management to comprehensive risk-based managed care organization in 2019. We excluded Arkansas 2016-2018 from analysis.

Source: Authors' analysis of the 2016-2019 Form CMS-416 data.

© 2023 Hu JC et al. *JAMA Network Open.*

eFigure. Sample Derivation Flowchart

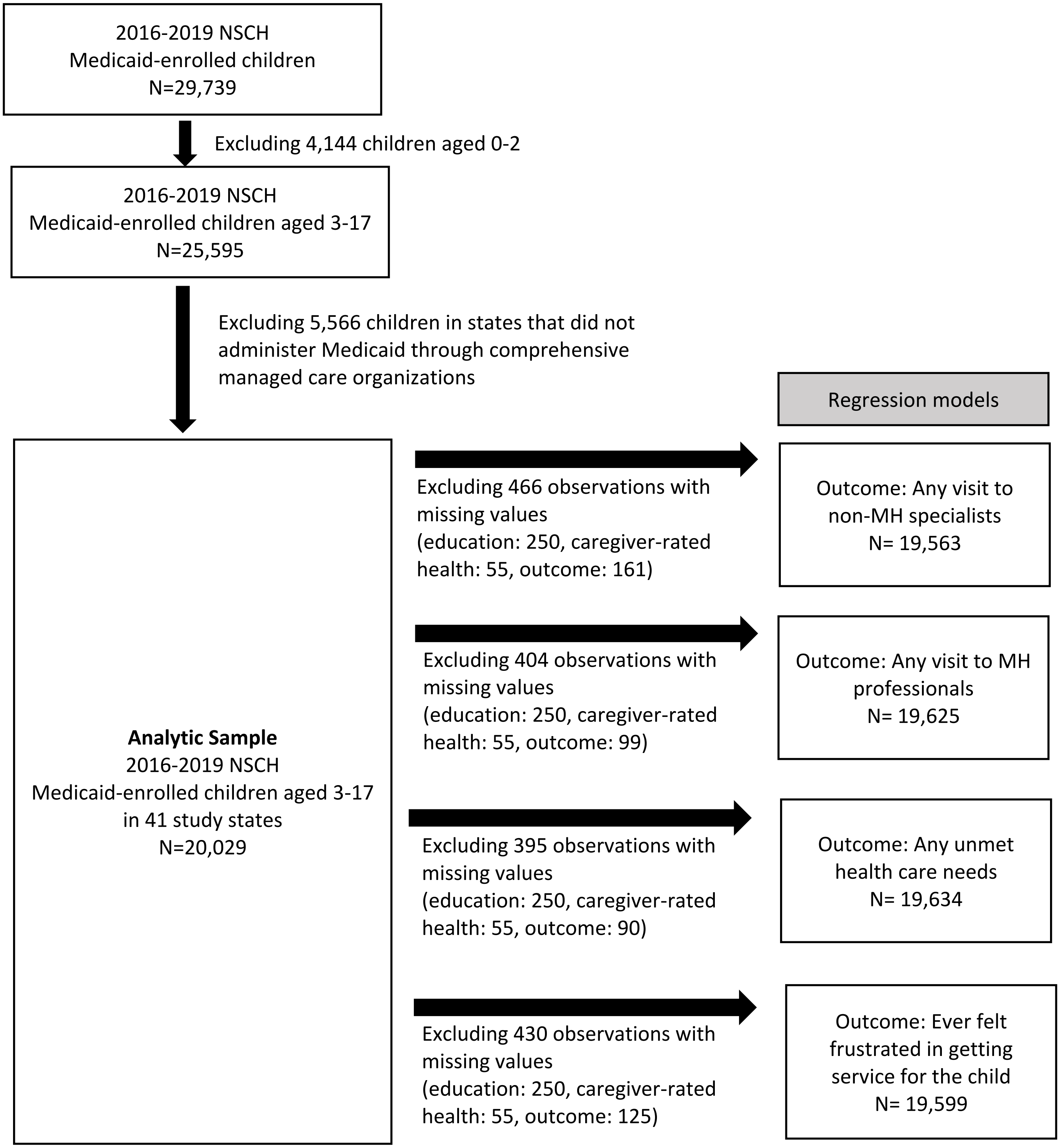

© 2023 Hu JC et al. *JAMA Network Open.*

eTable 2. Weighted and Unweighted Sample Size Across Study State-Years, 2016-2019

	Unweighted sample size					Weighted sample size (to be nationally representative)				
STATE	2016	2017	2018	2019	Total	2016	2017	2018	2019	Total
AR[a]	0	0	0	206	206	0	0	0	68,811	68,811
AZ	156	80	119	108	463	131,338	107,407	103,421	106,879	449,046
CA	177	88	115	123	503	790,374	633,278	697,902	636,613	2,758,168
CO	193	59	116	95	463	84,250	62,679	85,050	83,635	315,615
DC	153	73	88	77	391	9,862	9,619	9,839	8,377	37,696
DE	183	78	107	113	481	15,256	12,329	13,076	12,928	53,590
FL	221	114	172	140	647	314,838	367,423	368,893	316,412	1,367,566
GA	180	92	129	126	527	231,399	210,446	187,013	174,785	803,643
HI	175	87	106	86	454	15,756	17,754	15,160	14,150	62,820
IA	218	89	135	103	545	47,561	45,658	51,257	43,747	188,222
IL	189	78	99	112	478	191,505	188,590	207,128	221,298	808,520
IN	189	91	113	121	514	113,378	125,376	91,204	105,262	435,220
KS	173	65	96	98	432	47,493	37,131	40,780	45,389	170,794
KY	189	81	180	152	602	80,359	75,764	85,157	82,004	323,285
LA	219	120	178	176	693	116,306	105,382	95,129	98,996	415,813
MA	150	60	78	87	375	67,768	83,144	58,207	69,247	278,367
MD	160	71	86	88	405	75,663	71,645	87,104	76,019	310,433
MI	201	88	133	90	512	146,538	166,603	165,569	137,314	616,023
MN	209	65	78	81	433	74,546	70,614	75,721	63,545	284,425
MO	180	79	131	86	476	84,554	91,632	90,730	66,090	333,006
MS	202	167	204	209	782	76,940	84,348	68,272	68,017	297,577
ND	95	32	51	56	234	4,777	4,196	4,630	7,518	21,121
NE	130	57	71	65	323	22,790	21,082	23,590	20,038	87,500
NH	138	70	125	93	426	13,075	12,396	15,193	12,271	52,934
NJ	134	47	80	84	345	109,282	93,302	103,955	129,651	436,189
NM	232	127	205	209	773	48,245	53,081	48,653	53,723	203,701
NV	129	82	91	118	420	42,509	47,027	34,540	42,795	166,871
NY	186	105	104	153	548	292,306	347,860	251,704	330,736	1,222,606

© 2023 Hu JC et al. *JAMA Network Open.*

OH	176	70	116	107	469	180,440	170,261	170,947	178,698	700,347
OR	195	69	108	121	493	61,115	51,515	61,867	60,666	235,163
PA	219	83	135	141	578	188,515	204,342	197,067	174,095	764,019
RI	151	90	104	121	466	14,958	15,088	11,771	12,951	54,768
SC	212	103	135	160	610	95,215	101,675	86,640	101,606	385,137
TN	184	86	154	122	546	113,784	109,921	122,189	96,229	442,123
TX	155	73	125	96	449	523,169	559,797	529,805	464,140	2,076,912
UT	109	35	65	53	262	27,939	28,973	32,421	31,024	120,357
VA	133	65	86	70	354	86,456	116,685	108,359	87,962	399,463
VT	299	121	159	154	733	10,303	9,455	10,157	9,674	39,589
WA	224	69	116	117	526	120,688	93,472	104,896	106,405	425,461
WI	205	103	199	184	691	33,546	30,073	34,756	34,405	132,780
WV	169	54	86	92	401	67,604	68,606	62,469	74,286	272,965
Total	7,192	3,266	4,778	4,793	20,029	4,772,403	4,705,631	4,612,221	4,528,389	18,618,645

Note: [a]Arkansas switched its Medicaid program from primary care case management to comprehensive risk-based managed care organization in 2019. Study sample included Medicaid-enrolled children aged 3-17 years old in states that administered Medicaid program through comprehensive risk-based managed care organizations from 2016 to 2019.

© 2023 Hu JC et al. *JAMA Network Open.*
