# Supplementary material for: State-Level Variation in Medicaid Managed Care Enrollment and Specialty Care for Publicly Insured Children
Source: JAMA Netw Open. 2023 Oct 5;6(10):e2336415. doi: 10.1001/jamanetworkopen.2023.36415 (PMC10556966; doi:10.1001/jamanetworkopen.2023.36415)
Supplement: Supplement 2. — Data Sharing Statement [file jamanetwopen-e2336415-s002.pdf]

Data Sharing Statement

Hu. State-Level Variation in Medicaid Managed Care Enrollment and Specialty Care for Publicly Insured Children. *JAMA Netw Open*. Published October 05, 2023. doi:10.1001/jamanetworkopen.2023.36415

Data
Data available: No

Additional Information
Explanation for why data not available: The data that support the findings of this study are openly available in the National Survey of Children's Health, Form CMS-416, and Area Health Resources Files. These data were derived from the following publically available resources: 1. National Survey of Children's Health: https://www.childhealthdata.org/learn-about-the-nsch/NSCH 2. Form CMS-416: https://www.medicaid.gov/medicaid/benefits/early-and-periodic-screening-diagnostic-and-treatment/index.html 3. Area Health Resources Files: https://data.hrsa.gov/data/download
